# Targeting cyclophilin-D by miR-1281 protects human macrophages from *Mycobacterium tuberculosis*-induced programmed necrosis and apoptosis

**DOI:** 10.18632/aging.102593

**Published:** 2019-12-28

**Authors:** Qin Sun, Xiaona Shen, Peng Wang, Jun Ma, Wei Sha

**Affiliations:** 1 Clinic and Research Center of Tuberculosis, Shanghai Key Laboratory of Tuberculosis, Shanghai Pulmonary Hospital, Tongji University School of Medicine, Shanghai, China

**Keywords:** (MTB) *Mycobacterium tuberculosis*, macrophages, miR-1281, cyclophilin-D, programmed necrosis

## Abstract

*Mycobacterium tuberculosis* (MTB) infection induces cytotoxicity to host human macrophages. The underlying signaling mechanisms are largely unknown. Here we discovered that MTB infection induced programmed necrosis in human macrophages, causing mitochondrial cyclophilin-D (CypD)-p53-adenine nucleotide translocator type 1 association, mitochondrial depolarization and lactate dehydrogenase medium release. In human macrophages MTB infection-induced programmed necrosis and apoptosis were largely attenuated by CypD inhibition (by cyclosporin A), silencing and knockout, but intensified with ectopic CypD overexpression. Further studies identified microRNA-1281 as a CypD-targeting miRNA. Ectopic overexpression of microRNA-1281 decreased CypD 3’-untranslated region activity and its expression, protecting human macrophages from MTB-induced programmed necrosis and apoptosis. Conversely, microRNA-1281 inhibition in human macrophages, by the anti-sense sequence, increased CypD expression and potentiated MTB-induced cytotoxicity. Importantly, in CypD-KO macrophages miR-1281 overexpression or inhibition was ineffective against MTB infection. Restoring CypD expression, by an untranslated region-depleted CypD construct, reversed miR-1281-induced cytoprotection against MTB in human macrophages. Collectively, these results show that targeting CypD by miR-1281 protects human macrophages from MTB-induced programmed necrosis and apoptosis.

## INTRODUCTION

Macrophages, among other host immune cells, are essential in determining immune responses against *Mycobacterium tuberculosis* (MTB) infection and tuberculosis (TB) [1], which causes an estimated over 1.5 million human mortalities each year [2]. After activation, macrophages are capable of clearing the intracellular MTB burdens [1]. Contrarily, MTB bacteria can survive and then spread when the infected macrophages are dead [1, 3, 4]. Studies have shown that MTB spread will be facilitated with the death of the infected macrophages [1, 3, 4], caused often by the extracellular growth of released MTB or less cleared MTB in dead macrophages [3, 5]. Understanding the molecular mechanisms of death of MTB-infected macrophages is therefore important for MTB infection control [6].

Cell necrosis is traditionally known as a passive cell death form. Interestingly, recent literatures have indicated that cell necrosis could also be a programmed, mitochondria-dependent and active cell death [7–10]. This so-called “programmed necrosis” can promote cell death by a number of different stresses and stimuli, including oxidative injury, calcium over-load and several chemo-agents [7, 8, 11, 12]. In the progression of programmed necrosis, p53 translocates to cell mitochondria to form a complex with mitochondria permeability transition pore (mPTP) components, including cyclophilin-D (CypD) and adenine nucleotide translocator type 1 (ANT1) [13, 14]. This will lead to mitochondrial depolarization, mPTP opening and cytochrome C release. It will eventually promote cell necrosis [7–9, 11, 12, 15, 16]. Other studies proposed that the cascade is also important for initiating cell apoptosis, as cytochrome C releases to the cytosol [17–19]. The current study tested whether this pathway participated in MTB-induced death of human macrophages.

MicroRNAs (miRNAs) are a large family of endogenous, short (about 22-nt long) and single-strand non-coding RNAs (ncRNAs) [20, 21]. By physically binding to the 3′-untranslated region (3′-UTR) of the targeted mRNA, miRNAs will induce degradation of target mRNAs and/or inhibit gene translation [20, 21]. Existing literatures have implied that miRNA dysregulation in the host cells (including macrophages) is extremely important in active and latent TB infection [22–25]. Our previous study has shown that microRNA-579 (miR-579) upregulation mediated MTB-induced macrophage cytotoxicity [26]. Whether CypD is a target of miRNAs and the molecular regulation of CypD in the necrotic machinery of MTB-infected human macrophages remain to be elucidated. The results of the present study will show that microRNA-1281 (miR-1281) is a CypD-targeting miRNA, and miR-1281 protecting human macrophages from MTB-induced programmed necrosis and apoptosis by silencing CypD.

## RESULTS

### MTB infection induces mPTP opening and programmed necrosis in human macrophages

**Figure 1:**
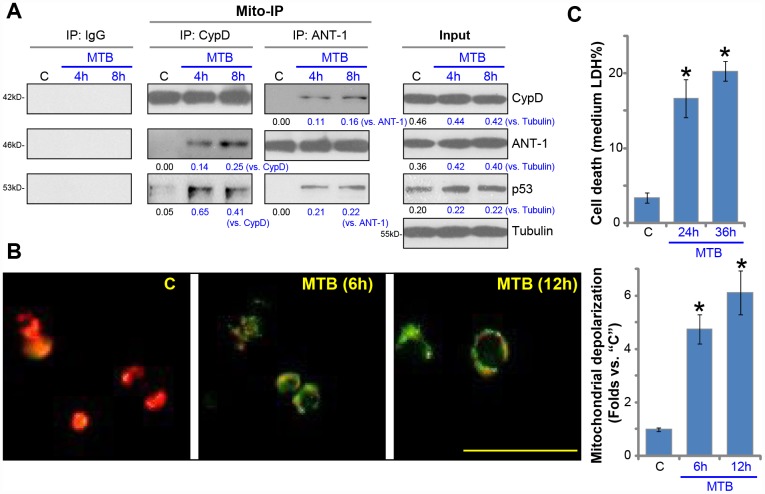
**MTB infection induces mPTP opening and programmed necrosis in human macrophages.** The primary human macrophages were infected with *Mycobacterium tuberculosis* (MTB) for applied time periods, mitochondrial immunoprecipitation (Mito-IP) assays were carried out to test CypD-ANT1-p53 association in the mitochondria (**A**, “Mito-IP”), with expression of these proteins examined by Western blotting (A, “Input”); Mitochondrial depolarization was examined by JC-1 dye assay (**B**); Cell necrosis was tested by medium LDH release assays (**C**). For JC-1 assays, both JC-1 merged images and JC-1 green fluorescence intensity were presented (same for all Figures). Expression of listed proteins was quantified, normalized to loading controls (**A**). “C” stands for uninfected control macrophages (same for all Figures). Data were presented as mean ± SD (n=5), and results were normalized to “C”. * *P* <0.05 vs. “C” macrophages. Experiments in this figure were repeated five times with similar results obtained. Bar= 100 μm (**B**).

Understanding the underlying mechanisms of MTB-induced death of macrophages is vital for the control of MTB infection [6, 26]. We tested the possible involvement of mPTP in the process. The mitochondrial immunoprecipitation (Mito-IP) assay results, Figure 1A, demonstrated that with MTB infection, p53 immunoprecipitated with mPTP components CypD and ANT1 [8, 27, 28]. It is known as the initial step for mPTP opening and programmed necrosis [11, 13, 14, 29, 30]. The expression levels of CypD, ANT1 and p53 were not significantly changed in human macrophages (Figure 1A, “Input”). mPTP opening is often followed with mitochondrial depolarization [11, 13, 14, 29, 30]. JC-1 assay results, Figure 1B, demonstrated that mitochondrial depolarization occurred in the MTB-infected human macrophages, showing JC-1 green fluorescence accumulation (Figure 1B). Furthermore, the medium LDH contents were significantly increased in human macrophages with MTB infection (Figure 1C), indicating programmed necrosis [11, 13, 14, 29, 30]. Together, these results suggested that MTB infection induced mPTP opening and programmed necrosis in human macrophages.

### CypD inhibition attenuates programmed necrosis and apoptosis in MTB-infected human macrophages

**Figure 2:**
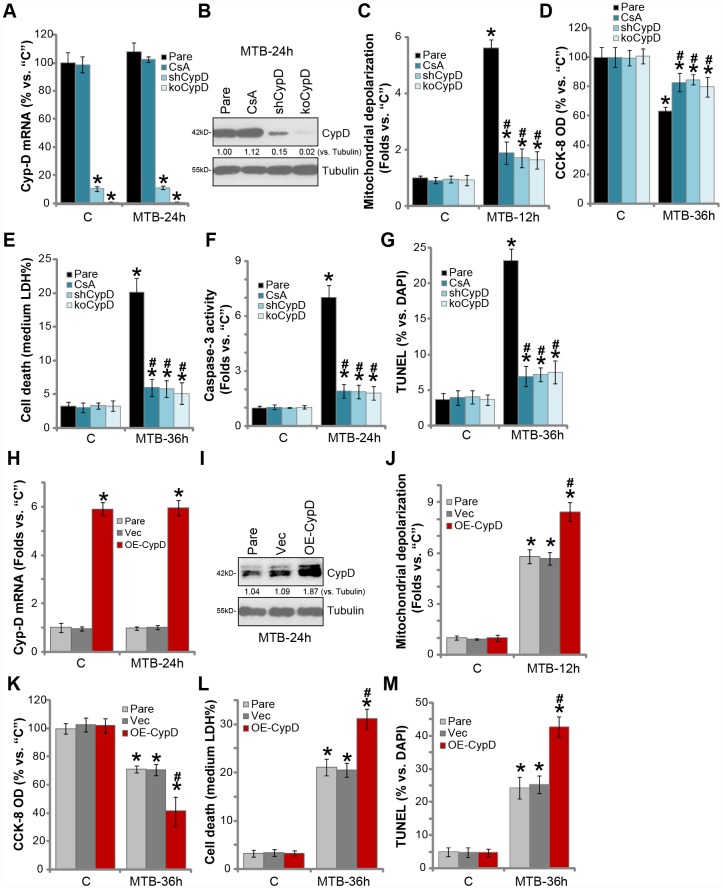
**CypD inhibition attenuates programmed necrosis and apoptosis in MTB-infected human macrophages.** The parental control human macrophages (“Pare”), with or without cyclosporin A (CsA) pretreatment (5 μM, for 1h), as well as the stale macrophages with the CypD shRNA (“shCypD”) or the lenti-CRISPR-Cas9 CypD knockout construct (“koCypD”), were infected with *Mycobacterium tuberculosis* (MTB) for applied time periods, *CypD mRNA* (**A**) and protein (**B**) expression was shown; Mitochondrial depolarization, cell viability, cell necrosis and apoptosis were tested by JC-1 staining (**C**), CCK-8 (**D**), medium LDH release (**E**), and Caspase-3/TUNEL assays (**F** and **G**) assays, respectively. The parental control human macrophages (“Pare”) as well as the stable macrophages with the CypD-expression construct (“OE-CypD”) or the empty vector (“Vec”) were infected with MTB for applied time periods, *CypD mRNA* (**H**) and protein (**I**) expression was shown; Mitochondrial depolarization (**J**), cell viability (**K**) and cell necrosis (**L**) were tested similarly. CypD expression was quantified, normalized to Tubulin (**B** and **I**). Data were presented as mean ± SD (n=5), and results were normalized to “C”. * *P* <0.05 vs. “C” treatment in “Pare” macrophages. ^#^ *P* <0.05 vs. MTB treatment in “Pare” macrophages. Experiments in this figure were repeated four times with similar results obtained.

The pharmacological and genetic strategies were applied to interfere CypD in human macrophages. Cyclosporin A (CsA), a CypD inhibitor and mPTP blocker [31–33], was utilized. Alternatively, the lentiviral shRNA strategy and the CRISPR-Cas9 gene-editing method were applied to knockdown and knockout (KO) CypD in human macrophages respectively (see Methods), resulting in depletion of CypD (Figure 2A and 2B). As shown, CsA, without changing CypD expression (Figure 2A and 2B), potently attenuated MTB-induced mitochondrial depolarization (JC-1 green fluorescence accumulation, Figure 2C). Consequently, the CypD inhibitor largely attenuated MTB-induced viability reduction (Figure 2D) and cell necrosis (medium LDH release, Figure 2E). Furthermore, in human macrophages with CypD shRNA or KO, MTB-induced mitochondrial depolarization (Figure 2C), viability reduction (Figure 2D) and cell necrosis (Figure 2E) were also largely attenuated. The caspase-3 activity and TUNEL staining assay results demonstrated that CypD inhibition (by CsA), silencing or KO potently alleviated MTB-induced caspase-3 activation (Figure 2F) and apoptosis (Figure 2G) in human macrophages. Therefore, CypD inhibition or silencing potently inhibited MTB-induced programmed necrosis and apoptosis in human macrophages.

We further hypothesized that ectopic overexpression of CypD should facilitate MTB-induced cytotoxicity of human macrophages. The lentiviral CypD expression construct was transduced to human macrophages. Via selection by puromycin the stable cells were established, showing over five-folds *CypD mRNA* expression (vs. vector control cells, Figure 2H). CypD protein levels were significantly increased as well (Figure 2I). Importantly, ectopic CypD overexpression potentiated MTB-induced mitochondrial depolarization (Figure 2J), viability reduction (Figure 2K) and medium LDH release (Figure 2L). TUNEL staining results demonstrated that CypD overexpression significantly enhanced MTB-induced apoptosis activation (Figure 2M). MTB infection did not affect CypD expression in human macrophages (Figure 2A and 2H). Without MTB infection, CypD inhibition, silencing, KO or overexpression did not affect the functions of human macrophages (Figure 2C–2G, 2J–2M). These results show that inhibition of the CypD-mPTP pathway largely attenuated MTB-induced death of human macrophages.

### microRNA-1281 is CypD-targeting miRNA in human macrophages

**Figure 3:**
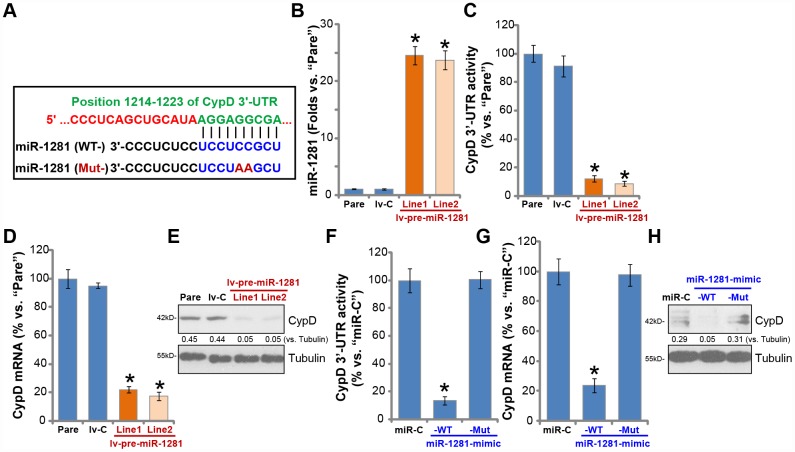
**microRNA-1281 is CypD-targeting miRNA in human macrophages.** microRNA-1281 (miR-1281) putatively targets position 1214-1223 in CypD 3’-UTR (3’-untranslated region) (**A**). The primary human macrophages were infected with lentivirus encoding pre-miR-1281 (“lv-pre-miR-1281”), two stable cell lines, “Line1/2”, were established following puromycin selection. Control macrophages were infected with non-sense microRNA (“lv-C”) lentivirus; Expression of mature miR-1281 and listed mRNAs was tested by qPCR assays (**B** and **D**); The relative CypD 3’-UTR activity was tested (**C**), with CypD protein expression tested by Western blotting (**E**). The primary human macrophages were transfected with 500 nM of control microRNA mimic (“miR-C”), the wild-type (“WT-”) or the mutant (“Mut-”) miR-1281 mimic (see sequences in A), after 48h CypD 3’-UTR activity (**F**), *CypD mRNA* (**G**) and protein (**H**) expression were tested. CypD protein expression was quantified, normalized to Tubulin (**E** and **H**). Data were presented as mean ± SD (n=5), and results were normalized. * *P* <0.05 vs. “Pare”/”miR-C” cells. Experiments in this figure were repeated five times with similar results obtained.

miRNAs are a large family of conserved, short single-stranded non-coding RNAs (ncRNAs), function as the negative regulators of the target genes by suppressing mRNA translation and/or promoting mRNA degradation [34, 35]. We have shown that CypD inhibition or silencing protected human macrophages from MTB infection. Therefore CypD-targeting miRNAs should exert similar functions. The possible CypD-targeting miRNAs were searched by consulting the microRNA database TargetScan (V7.2) at its 3’-UTR. The potential CypD-targeting miRNAs were further verified by other microRNA databases, including miRbase (v21.0), miRDB, miRanda and PicTar. These bioinformatics studies discovered that microRNA-1281 (miR-1281) putatively targets the 3’-UTR of CypD (at position 1214-1223, Figure 3A), with the miR-1281-CypD binding context score percentage of 99% and the context^++^ score -0.6 (from TargetScan V7.2, Figure 3A) [36].

To test whether miR-1281 could target and inhibit CypD expression, the lentiviral construct with pre-miR-1281 (“lv-pre-miR-1281”) was transduced to human macrophages. Following selection two stable cell lines, “Line1/Line2”, were established, where mature miR-1281 levels increased over 20 folds (vs. parental control macrophages, Figure 3B). Significantly, ectopic miR-1281 overexpression potently inhibited the 3’-UTR activity of CypD (Figure 3C). *CypD mRNA* levels decreased over 80% in lv-pre-miR-1281-expressing macrophages (vs. control macrophages, Figure 3D), where CypD protein levels were significantly downregulated as well (Figure 3E).

To further show that miR-1281 is a CypD-targeting miRNA, the primary human macrophages were transfected with wild type (“WT-”) or a mutant (“Mut-”) miR-1291 mimic. The Mut-miR-1291 mimic does not bind to the 3′-UTR of CypD (Figure 3A). Transfection of the WT-miR-1291 mimic led to significant reduction of CypD 3′-UTR activity (Figure 3F) and *CypD mRNA*/protein expression (Figure 3G and 3H), with the Mut-miR-1291 mimic completely ineffective (Figure 3F–3H). These results suggest that miR-1281 is a CypD-targeting miRNA in human macrophages.

### miR-1281 overexpression inhibits MTB-induced programmed necrosis and apoptosis in human macrophages

**Figure 4:**
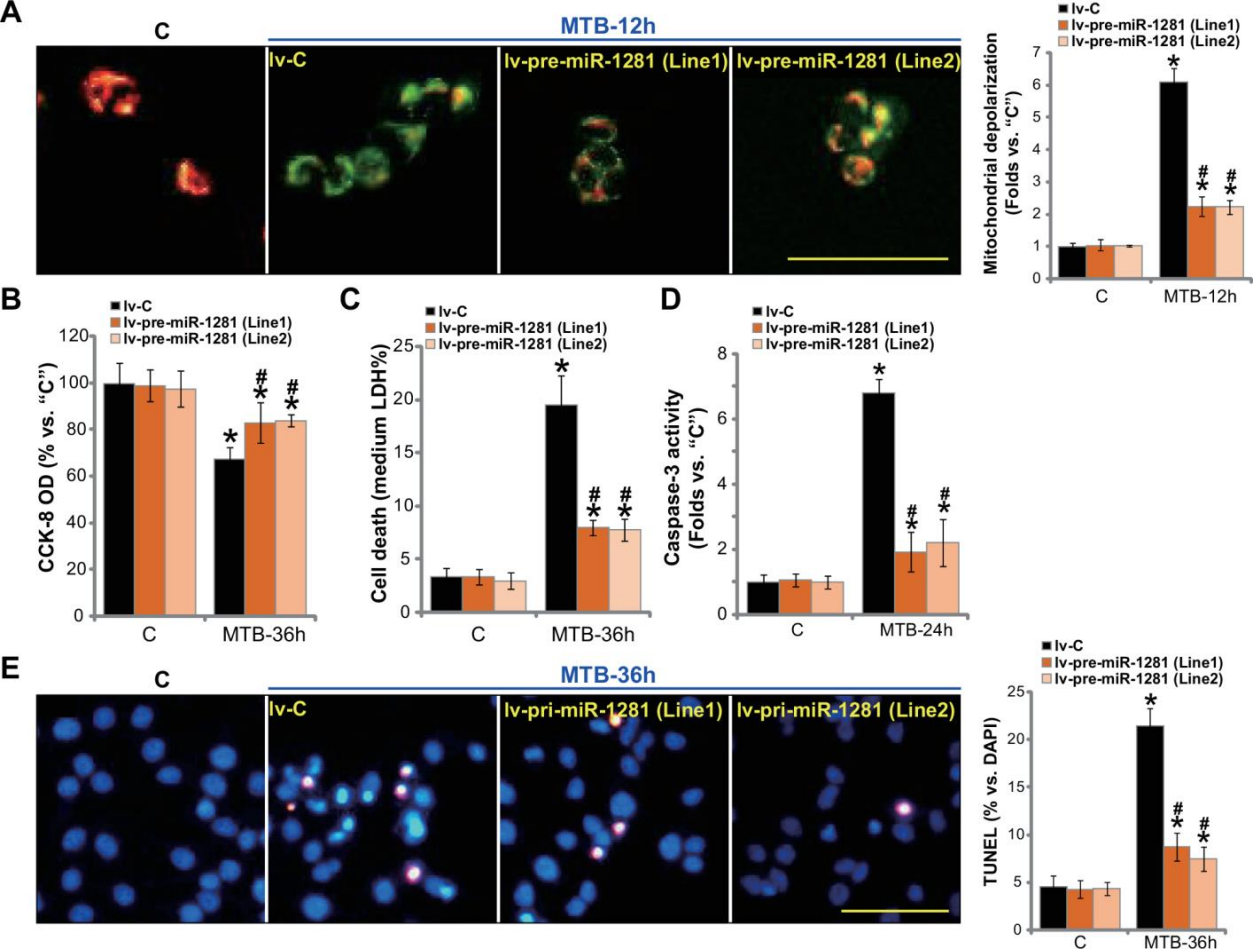
**miR-1281 overexpression inhibits MTB-induced programmed necrosis and apoptosis in human macrophages.** The primary human macrophages were infected with lentivirus encoding pre-miR-1281 (“lv-pre-miR-1281”), two stable cell lines, “Line1/2”, were established following puromycin selection. Control macrophages were infected with non-sense microRNA (“lv-C”); The macrophages were infected with *Mycobacterium tuberculosis* (MTB) for applied time periods, mitochondrial depolarization, cell viability, cell necrosis and apoptosis were tested by JC-1 staining (**A**), CCK-8 (**B**), medium LDH release (**C**), and caspase-3 activity (**D**)/TUNEL staining (**E**) assays, respectively. Data were presented as mean ± SD (n=5). * *P* <0.05 vs. “C” treatment in “lv-C” macrophages. ^#^ *P* <0.05 vs. MTB treatment in “lv-C” macrophages. Experiments in this figure were repeated five times with similar results obtained. Bar= 100 μm (**A** and **E**).

Since miR-1281 targets and downregulates CypD, it would then protect human macrophages from MTB-induced cytotoxicity. The lv-pre-miR-1281-expressing human macrophages (see Figure 3) and control macrophages with non-sense microRNA (“lv-C”) were infected with MTB. As shown, miR-1281 overexpression potently inhibited MTB-induced mitochondrial depolarization, or JC-1 green fluorescence accumulation (Figure 4A). MTB-induced viability reduction (Figure 4B) and cell necrosis (medium LDH release, Figure 4C) were significantly attenuated in lv-pre-miR-1281-expressing macrophages. Furthermore, miR-1281 overexpression in human macrophages suppressed MTB-induced caspase-3 activation (Figure 4D) and apoptosis (nuclear TUNEL staining, Figure 4E). Thus, miR-1281 overexpression alleviated MTB-induced programmed necrosis and apoptosis in human macrophages.

### miR-1281 inhibition upregulates CypD and intensifies MTB-induced cytotoxicity in human macrophages

**Figure 5:**
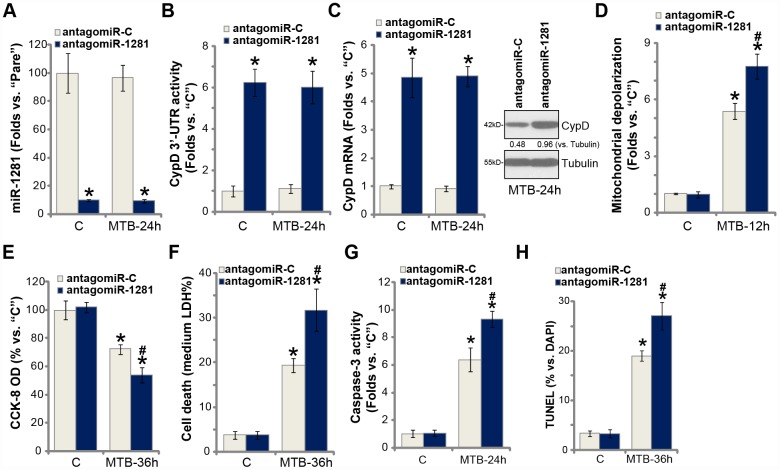
**miR-1281 inhibition upregulates CypD and intensifies MTB-induced cytotoxicity in human macrophages.** The primary human macrophages were transduced with the lentiviral pre-miR-1281 anti-sense (“antagomiR-1281”) or the non-sense control miR anti-sense (“antagomiR-C”), with stable cells selected by puromycin. Macrophages were then infected with *Mycobacterium tuberculosis* (MTB) for applied time periods, expression of mature miR-1281 and listed *genes* was shown (**A** and **C**); The relative CypD 3’-UTR activity was tested (**B**); Mitochondrial depolarization, cell viability, cell necrosis and apoptosis were tested by JC-1 staining (**D**), CCK-8 (**E**), medium LDH release (**F**), and caspase-3 activity (**G**)/TUNEL staining (H) assays, respectively. CypD expression was quantified, normalized to Tubulin (**C**). Data were presented as mean ± SD (n=5). * *P* <0.05 vs. “C” treatment in “antagomiR-C” macrophages. ^#^ *P* <0.05 vs. MTB treatment in “antagomiR-C” macrophages. Experiments in this figure were repeated five times with similar results obtained.

To suppress miR-1281 expression, the lentivirus encoding the pre-miR-1281 anti-sense sequence, antagomiR-1281, was transduced to the primary human macrophages, resulting in over 90% reduction of mature miR-1281 expression (Figure 5A). Conversely, CypD 3′-UTR activity (Figure 5B), *CypD mRNA* (Figure 5C, the left panel) and protein (Figure 5C, the right panel) expression were significantly elevated (Figure 5B, 5C). Functional studies demonstrated that miR-1281 inhibition intensified MTB-induced mitochondrial depolarization (Figure 5D). As compared to control macrophages (with anti-sense control sequence/ antagomiR-C), the macrophages with antagomiR-1281 showed increased viability reduction (Figure 5E), cell necrosis (Figure 5F) and apoptosis (Figure 5G and 5H) following MTB infection. Therefore, miR-1281 inhibition upregulated CypD and intensified MTB-induced cytotoxicity in human macrophages.

### miR-1281 is ineffective in CypD-depleted human macrophages

**Figure 6:**
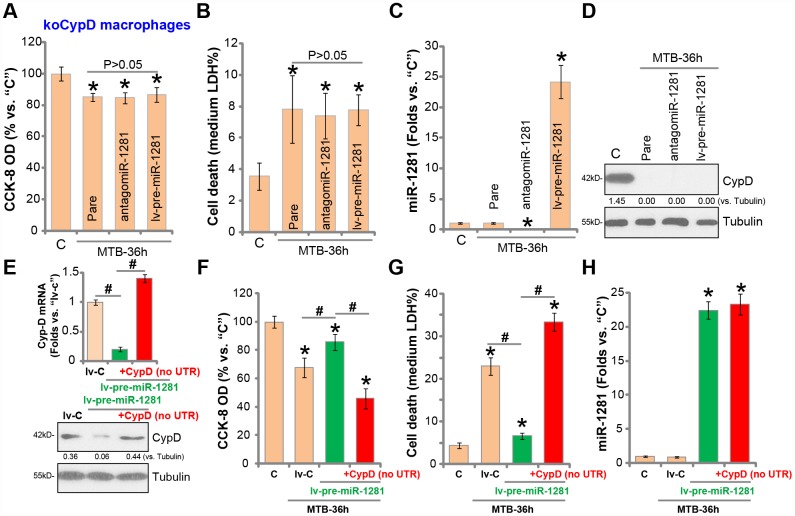
**miR-1281 is ineffective in CypD-depleted human macrophages.** The stale human macrophages with the lenti-CRISPR-Cas9 CypD knockout construct (“koCypD”) were infected with the lentivirus encoding pre-miR-1281 (“lv-pre-miR-1281”) or antigomiR-1281, with puromycin selection stable cells were established. The macrophages were then treated with *Mycobacterium tuberculosis* (MTB) infection for applied time periods, cell viability (CCK-8 assay, **A**), cell necrosis (medium LDH release assay, **B**), miR-1281 levels (**C**) and CypD protein expression (**D**) were tested. The stale macrophages with lv-pre-miR-1281 were further transfected with the construct encoding the 3’UTR-depleted CypD (“no UTR”), after 48h *CypD mRNA* and protein expression was tested (**E**); The macrophages were further infected with MTB for applied time periods, cell viability (**F**), cell necrosis (**G**) and miR-1281 expression (H) were examined. CypD expression was quantified, normalized to Tubulin (D and E). Data were presented as mean ± SD (n=5). * *P* <0.05 vs. “C” treatment. ^#^ *P* <0.05 (**E** and **G**). Experiments in this figure were repeated five times with similar results obtained.

If CypD is the target of miR-1281, the latter should be ineffective in the CypD-depleted cells. Therefore, to the CypD-KO human macrophages (see Figure 2), lv-pre-miR-1281 or antigomiR-1281 was transduced. As shown lv-pre-miR-1281 and antigomiR-1281 failed to affect MTB infection-induced viability reduction (Figure 6A) and cell necrosis (Figure 6B) in CypD-KO macrophages. Both altered miR-1281 expression (Figure 6C). Western blotting assay results, Figure 6D, confirmed that CypD depletion in the CypD-KO human macrophages.

Next, the 3′-UTR-depleted CypD construct was transfected to human macrophages, completely restored *CypD mRNA* and protein expression in macrophages with lv-pre-miR-1281 (Figure 6E). As shown lv-pre-miR-128-induced macrophage protection against MTB was completely reversed with re-expression of the 3′-UTR-depleted CypD (Figure 6F and 6G). Thus, with CypD re-expression MTB-induced viability reduction (Figure 6F) and cell death (Figure 6G) were restored even with miR-1281 overexpression. The qPCR assay results, Figure 6H, demonstrated that 3′-UTR-depleted CypD did not alter miR-1281 expression. These results together indicate that CypD should be the important target of miR-1281 in human macrophages.

## DISCUSSION

Necrosis is a common form of cell death characterized by cell swelling, plasma membrane fracture and lysis of the intracellular components and cellular organelles. The traditional concept is that necrosis is a form of accidental, unregulated and passive cell death, while apoptosis is the sole form of “programmed cell death” [10, 37, 38]. Yet recent studies have shown that certain necrosis is also programmed and actively regulated [7, 10, 37–39]. In the present study we show that MTB infection led to programmed necrosis in human macrophages, causing CypD-p53-ANT1 mitochondrial association, mitochondrial depolarization and LDH release (to the medium). Importantly programmed necrosis, together with apoptosis, could be vital for MTB infection-induced cytotoxicity in the human macrophages.

CypD is the prolyl isomerase and the key component forming mPTP, along with ANT1 and the voltage-dependent anion channel (VDAC) [13, 14, 29]. Studies have shown that CypD lies in the center to mediate the pore opening. CypD inhibition or depletion will result in inhibition on mPTP formation and opening [13, 14, 29]. Since mPTP opening is vital for programmed necrosis, CypD is essential in regulating necrotic cell death pathway [13, 14, 29]. In the present study we show that CypD is vital for MTB-induced cytotoxicity to human macrophages. MTB infection-induced programmed necrosis and apoptosis were largely attenuated with CypD inhibition (by CsA), silencing (by shRNA) and KO (using CRISPR/Cas9 method), but intensified with ectopic overexpression of CypD. Therefore, targeting CypD-mPTP pathway could be a novel strategy to protect human macrophages from MTB infection-induced cytotoxicity.

One strategy to inhibit CypD-mPTP pathway is to express CypD-targeting miRNAs. Wang et al*.,* have shown that microRNA-30b (miR-30b) targeting CypD protected hearts from ischemia/reperfusion injury and necrotic cell death [40]. miR-7 also targets VDAC1 to shut down the function of mPTP pore [41]. The results of this study show that miR-1281 is an anti-CypD miRNA. Ectopic overexpression of miR-1281, by lv-pre-miR-1281, significantly decreased CypD 3′-UTR activity and downregulated *CypD mRNA*/protein expression in human macrophages. Conversely, miR-1281 inhibition, by antagomiR-1281, led to increased CypD 3′-UTR activity and expression. The mutant miR-1281, with the mutation at the CypD 3′-UTR binding site, failed to alter CypD 3′-UTR activity and expression. These results clearly show that miR-1281 targets CypD in human macrophages.

Our results imply that miR-1281 inhibited MTB-induced cytotoxicity to the human macrophages. First, lv-pre-miR-1281 largely attenuated programmed necrosis and apoptosis in MTB-infected macrophages. Conversely, miR-1281 inhibition, by antagomiR-1281, protected human macrophages from MTB-induced cytotoxicity. These results imply that miR-1281 offers cytoprotection against MTB infection in human macrophages. Further analyses show that CypD is the primary target gene of miR-1281 in MTB-infected macrophages. Neither miR-1281 overexpression nor miR-1281 inhibition was able to change MTB-induced cytotoxicity in CypD-KO macrophages. Importantly, restoring CypD expression, by the UTR-depleted CypD construct, reversed miR-1281-induced macrophage protection against MTB infection.

Collectively, these results show that targeting CypD by miR-1281 protects human macrophages from MTB-induced programmed necrosis and apoptosis.

## MATERIALS AND METHODS

### Chemicals and reagents

Puromycin, cyclosporin A (CsA), terminal deoxynucleotidyl transferase (TdT)-mediated Dutp nick-end labeling (TUNEL), DAPI and JC-1 dyes were obtained from Sigma-Aldrich (St. Louis, MO). The antibodies were from Cell Signaling Tech (Danvers, MA). From Invitrogen-Thermo Fisher (Shanghai, China) the cell culture reagents, the Trizol reagents and other RNA assay reagents, as well as the cell transfection reagents were obtained. All the sequences, viral constructs and gene products were provided and verified by Shanghai Genechem Co. (Shanghai, China) or otherwise mentioned.

### Primary human macrophages.

As described early [26], from the peripheral blood mononuclear cells (PBMCs) of a written-informed consent donor the primary human macrophages were differentiated [42] and cultured under the described protocol [42]. The primary macrophages were always utilized at passage 3–10. The protocols of the present study were approved by the Ethics Committee of Tongji University School of Medicine.

### MTB infection

As described early [26], at 2×10^5^ cells per well the primary human macrophages were cultured into six-well plates and then infected with MTB (multiplicity of infection/MOI 10). After 4h the infected macrophages were washed and returned back to the fresh medium.

### Mitochondrial Immunoprecipitation (Mito-IP)

As described previously [18], human macrophages with MTB infection were harvested and homogenized by the lysis buffer provided by Dr. Wang at Soochow University [18]. After centrifugation, the supernatants were collected and suspended. The pellets were then re-suspended in the above buffer plus NP-40, forming the mitochondria fraction lysates. The quantified mitochondrial lysates (500 μg per sample) were pre-cleared and incubated with anti-CypD antibody [28, 43], with the mitochondrial CypD-p53-ANT1 complex captured by the protein IgG-Sepharose beads (Sigma), and tested by Western blotting.

### Quantitative real-time PCR (qPCR)

Total cellular RNA was extracted by the Trizol reagents from MTB-infected macrophages, with the RNA concentrations determined using the NanoDrop system. From each treatment 100 ng total RNA was utilized for the reverse transcription using the described protocol [26]. The detailed procedures for qPCR were described previously [26], with the melt curve analyses performed. Quantification of targeted genes was through the 2^−ΔΔCt^ method, using GAPDH as the internal control. miR-1281 expression was normalized to *U6.* From Shanghai Genechem the primers for *U6* and *GAPDH* were obtained, with other primers for miR-1281, *CypD* and *ANT1* listed in Table 1.

**Table 1:** Primers utilized in this study.

**Gene name**	**Forward primer**	**Reverse primer**
miR-1281	TCGCCTCCTCCTCTCC	GAACATGTCTGCGTATCTC
ANT-1	GCTGCCTACTTCGGAGTCTATG	TGCGACTGCCGTCACACTCTG
CypD	CGACTTCACCAACCACAATGGC	GGTGTTAGGACCAGCATTAGCC

### Western blotting

The detailed procedures for the Western blotting assay were reported early [26]. In brief, with the applied treatments, 30 μg total lysates (of each lane) were separated by sodium dodecyl sulfate-polyacrylamide gels, thereby transferred to the polyvinylidene difluoride (PVDF) blots (Merck-Millipore). After blocking the blots were incubated with the primary and secondary antibodies, and detected using the enhanced chemiluminescence (ECL) kit (Pierce, Rockford, IL).

### Cell viability

Macrophages were plated at 3×10^3^ cells per well onto the 96-well tissue-culture plates. Following the indicated treatments the Cell Counting Kit-8 (CCK-8, Dojindo Laboratories, Kumamoto, Japan) reagent (10 μL in each well) was added. After 2h, the CCK-8 absorbance at 450 nm was tested through a spectrophotometer (Thermo Fisher Scientific, Vantaa, Finland).

### Cell necrosis

Cell necrosis was tested through assaying the medium lactate dehydrogenase (LDH) contents by a two-step easy enzymatic reaction LDH kit (Takara, Tokyo, Japan). Medium LDH contents were always normalized to total LDH levels.

### TUNEL staining

Following MTB infection, the human macrophages were co-stained with TUNEL and DAPI dyes (Sigma). The apoptotic nuclei percentage (TUNEL/DAPI×100%) was calculated, from at least 500 cells of five random views (1: 100 magnification).

### Caspase-3 activity

The caspase-3 activity was examined by the commercial kit (Promega, Shanghai, China). After treatment, 20 μg of cytosol extracts (per treatment) was added to the caspase assay buffer (Beyotime, Wuxi, China). The release of 7-amido-4-(trifluoromethyl) coumarin (AFC) was quantified via the Fluoroskan system (Thermo-Labsystems, Helsinki, Finland) at the test-wavelength of 535 nm.

### JC-1 assay

As described previously [26], the human macrophages with the indicated treatment were stained with JC-1 (5 μg/mL, for 10-15 min) and washed. JC-1 green fluorescence, indicating mitochondrial depolarization, was tested at 550 nm using the RF-5301 PC fluorescence spectrofluorometer (Shimadzu, Tokyo, Japan). Furthermore, the representative JC-1 fluorescence images were taken, merging the green fluorescence image (at 550 nm) and the corresponding red fluorescence image (at 650 nm).

### miR-1281 overexpression and inhibition

The protocols were described previously [26]. In brief, the pre-miR-1281 sequence and the pre-miR-1281 anti-sense sequence were synthesized, sequence-verified (both from Shanghai Genechem) and individually ligated into the GV248 lentiviral construct [26]. The construct was transfected to HEK-293T cells together with the lentivirus package plasmids (Shanghai Genechem) [44]. After 48h, the pre-miR-1281-expressing lentivirus (“lv-pri-miR-1281”) or pre-miR-1281 anti-sense lentivirus (“antagomiR-1281”) were obtained, enriched (MOI at 20), filtered and added to human macrophages, cultured in polybrene-containing complete medium and selected by puromycin to achieve stable cells, with miR-1281 levels tested by qPCR.

### CypD 3′-UTR luciferase activity assay

The CypD 3′-UTR reporter plasmid (pMIR-REPORT plasmid, containing the miR-1281-binding sites, at position 1214-1223, generated by Shanghai Genechem) was transfected to human macrophages using the Lipofectamine 2000 (Invitrogen Thermo-Fisher, Shanghai, China) protocol. The transfected macrophages were then subjected to the applied genetic treatments, with the 3'-UTR luciferase activity tested by the Promega kit [45].

### CypD short hairpin RNA (shRNA)

The CypD shRNA (with the target sequence, CCCG TCCTCTTCCTCCTCCTCCG) lentiviral particles and the control shRNA lentiviral particles were provided by Dr. Xu [46]. Human macrophages were plated onto six-well plates (in polybrene-containing complete medium), transduced with the applied shRNA lentivirus particles. After 48h, puromycin was added to select stable cells (for 10–12 days), with CypD silencing verified by qPCR and Western blotting assays.

### CypD knockout (KO)

The small guide RNA (sgRNA) against human CypD (target DNA sequence, GGCGACTTCACCAACCA CAA) was selected from Dr. Zhang’s laboratory (http://crispr.mit.edu/), and inserted into the lentiCRISPR-green fluorescent protein (GFP) plasmid (from Dr. Zhao at Shanghai Jiao Tong University) with the puromycin selection gene. The construct was transfected to the human macrophages by Lipofectamine 2000, with macrophages subjected to FACS-mediated GFP sorting and selected by puromycin (3.0 μg/mL) to achieved stable cells. CypD KO was verified by qPCR and Western blotting assays. Control cells were transfected with the empty vector.

### Ectopic CypD over-expression.

The CypD expression (with no 3′-UTR region) pSuper-puro-Flag vector, provided by Dr. Xu [46], was transfected to human macrophages by the Lipofectamine 2000 protocol (Invitrogen, Suzhou, China). The macrophages were then selected by puromycin for 10 days to achieve stable cells, with CypD overexpression confirmed by qPCR and Western blotting assays.

### Statistical analyses

Data in the present study were shown as mean ± standard deviation (SD). Statistical analyses were carried out by the SPSS 20.0 software (SPSS Co., Chicago, CA), using one-way analysis of variance of post hoc Bonferroni test as comparisons of multiple groups. The Student T Test was utilized for comparison between two groups. Statistically differences were assigned to *P* < 0.05.
